# A NiFe Alloy Reduced on Graphene Oxide for Electrochemical Nonenzymatic Glucose Sensing

**DOI:** 10.3390/s18113972

**Published:** 2018-11-15

**Authors:** Zhe-Peng Deng, Yu Sun, Yong-Cheng Wang, Jian-De Gao

**Affiliations:** 1 College of Chemistry and Chemical Engineering, Northwest Normal University, Lanzhou 730070, China; dengzp516@163.com; 2 Gansu Computing Center, Lanzhou 730030, China; 3 Experiment Center of Northwest University for Nationalities, Lanzhou 730030, China; sunyu794@163.com; 4 College of Pharmacy, Gansu University of Chinese Medicine, Lanzhou 730000, China; 13919124704@163.com

**Keywords:** NiFe alloy, graphene oxide, glucose, nonenzymatic sensor

## Abstract

A NiFe alloy nanoparticle/graphene oxide hybrid (NiFe/GO) was prepared for electrochemical glucose sensing. The as-prepared NiFe/GO hybrid was characterized by transmission electron microscopy (TEM) and X-ray diffraction (XRD). The results indicated that NiFe alloy nanoparticles can be successfully deposited on GO. The electrochemical glucose sensing performance of the as-prepared NiFe/GO hybrid was studied by cyclic voltammetry and amperometric measurement. Results showed that the NiFe/GO-modified glassy carbon electrode had sensitivity of 173 μA mM^−1^ cm^−2^ for glucose sensing with a linear range up to 5 mM, which is superior to that of commonly used Ni nanoparticles. Furthermore, high selectivity for glucose detection could be achieved by the NiFe/GO hybrid. All the results demonstrated that the NiFe/GO hybrid has promise for application in electrochemical glucose sensing.

## 1. Introduction

Glucose sensing is important in many fields, such as medical diagnostics and the food industry [1]. The earliest glucose sensing was reported by Clark and Lyons in 1962, using a biological enzyme [2]. Since then, biological enzymes such as glucose oxidase and glucose dehydrogenase have been widely used for glucose sensing. However, biological enzymes are susceptible to external conditions such as temperature, humidity, and pH, among others, which lead to the instability of the biological enzymes [3,4,5]. The expensive price and complicated mobilizing methods of biological enzymes also restrict their application [6]. Therefore, to address these problems, the research focus has been transferred to the development of electrochemical nonenzymatic glucose sensors.

Due to its high stability and sensitivity, nonenzymatic electrochemical technology is a good choice for glucose sensing [7]. Various nanoparticles have been reported for constructing electrochemical nonenzymatic biosensors, such as noble metal Pt nanoparticles (Pt NPs) [3,8,9], Au NPs [10,11], and Pd NPs [12,13]. However, the expensive price of these noble metals restricts their practical application. Furthermore, noble metal-constructed electrochemical glucose sensors are usually poisoned by chloride ions present in the body’s blood [14]. In order to exploit cheap electrochemical nonenzymatic glucose biosensors, considerable attention has been focusing on non-noble metal materials, such as Ni NPs [15,16,17], Co(OH)_2_ nanotubes [18], and Cu NPs [19,20]. Recent studies reveal that bimetallic materials [21], especially bimetal alloys, exhibit better catalytic performance than that of monometallic counterparts [22]. Xu et al. and Chen et al. both found that a Pt–Ni alloy exhibited enhanced sensitivity for glucose sensing [23,24]. Besides, a PtRu alloy [25], PtAu alloy [26], PtCo alloy [27], CoCu alloy [28], and NiCo alloy [29] have been used in glucose sensing.

A support material is also important in electrochemical glucose sensors [30]. Carbon-based materials (especially carbon nanotubes and graphene) have been extensively used to load the electrochemical catalyst due to their excellent conductivity, such as in loading cupric oxide [31] or growing copper nanoparticles [32] on carbon nanotubes (CNTs) for electrochemical glucose sensing. Graphene and its oxide (graphene oxide, GO) have triggered researchers’ interest since 2004 because of their good electrical conductivity and large specific surface area. Due to these exceptional properties, graphene and GO have often been chosen as the supporting material in electrochemistry [33]. GO loaded with NiS [34], NiCo alloy [29], and CuS NPs [35] have been applied for electrochemical glucose sensing.

Based on the above considerations, we tried to study the alloying of commonly used Ni NPs in the scope of glucose detection with Feon GO, because the NiFe alloy shows high electrochemical performance in various processes [36,37,38,39]. To the best of our knowledge, the NiFe alloy nanoparticle/graphene oxide hybrid (NiFe/GO) has never been reported for electrochemical glucose sensing. The NiFe/GO hybrid reveals better electrocatalytic performance towards glucose oxidation than the commonly used Ni NPs. The high performance of NiFe alloy nanoparticles and the superior conductivity of GO endow the NiFe/GO biosensor with the capacity to achieve the electrochemical sensing of glucose in a wide concentration range.

## 2. Materials and Methods

### 2.1. Chemicals and Reagents

All reagents used in experiments were of analytical grade. Graphite powder was purchased from Alfa Aesar (Shanghai, China). Nickel sulfate hexahydrate, iron sulfate heptahydrate, hydrazine hydrate (80 wt%), and glucose were procured from Sinopharm Chemical Reagent Co., Ltd. (Shanghai, China). Ascorbic acid (AA), uric acid (UA), and dopamine (DA) were purchased from Acros (Shanghai, China). Deionized water (18.2 MΩ·cm) was used in all experiments.

### 2.2. Preparation of GO

GO was prepared by a modified Hummers’ method [40]. Two grams of graphite and 1 g NaNO_3_ were mixed with 50 mL H_2_SO_4_ (95%) in a 250 mL flask within an ice bath to keep a low temperature (note that the ice bath is important and necessary). Then, 6 g KMnO_4_ was added slowly into the above suspension with vigorous stirring. In this adding process, the reaction temperature was kept below 20 °C. After that, the mixture was stirred at room temperature overnight. Subsequently, 60 mL H_2_O was added slowly with vigorous agitation. The reaction temperature was increased rapidly up to 95 °C, and the color of the suspension changed to yellow. Then, 10 mL of 30% H_2_O_2_ was added to the mixture. Finally, the obtained product was washed by rinsing with 5% HCl and then deionized water for several times until the pH of filtrate reached 7. After drying in a vacuum dryer, GO as a gray powderwas obtained.

### 2.3. Preparation of the NiFe/GO Composite

For the preparation of the NiFe/GO composite (for example, the ratio of Ni to Fe is 1:1, denoted as NiFe/GO), 0.1 g of the as-prepared GO, 278.1 mg FeSO_4_·7H_2_O (1 mmol), and 262.9 mg NiSO_4_·6H_2_O (1 mmol) were added to 10 mL deionized water. The mixture was sonicated for 30 min to get an evenly dispersed solution. Then, 15 mL hydrazine hydrate was dropped slowly into the above solution followed by refluxing at 100 °C for 3 h under N_2_ atmosphere. After reaction, the obtained NiFe/GO composite was washed using deionized water for several times. Finally, the washed NiFe/GO composite was dried in a vacuum for further use. Other composites (different Ni-to-Fe mass ratios, such as Ni_1_Fe_4_/GO, Ni_4_Fe_1_/GO, Ni/GO, and Fe/GO) were prepared by adjusting the ratio of Ni and Fe. NiFe alloy NPs were also prepared for comparison using the same method without adding GO.

### 2.4. Preparation of the NiFe/GO Hybrid Modified Electrode

Five milligrams of NiFe/GO hybrid was dispersed in 1 mL of solution containing 0.5 mL ethyl alcohol and 0.5 mL deionized water. The above solution was sonicated for 30 min to get an evenly dispersed mixture. After sonicating, a certain amount of the mixture was dropped onto a bare glassy carbon electrode (GCE), followed by adding 2 μL Nafion solution (0.5%) to entrap the NiFe/GO. The prepared electrode was denoted as NiFe/GO/GCE. For comparison, Ni/GO/GCE, Fe/GO/GCE, and NiFe/GCE were fabricated similarly.

### 2.5. Apparatus and Measurements

The morphologies of the NiFe/GO composite were collected with a G^2^F^30^ electron microscope (Tecnai, Hillsboro, OR, USA). XRD data were conducted on a D/max-2400 diffractometer (Rigaku, Beijing, China ) operating at a voltage of 40 kV and a current of 40 mA, using Cu-K radiation as the X-ray source. Electrochemical characterization was performed on a CHI 660C electrochemical workstation (CH Instruments Ins, Shanghai, China) with the modified GCE as the working electrode, Pt wire as the counter electrode, and Ag/AgCl (3 M KCl) as the reference electrode. All voltages used in the manuscript refer to the Ag/AgCl (3 M KCl) electrode.

## 3. Results and Discussion

### 3.1. Structural Characterization

**Figure 1:**
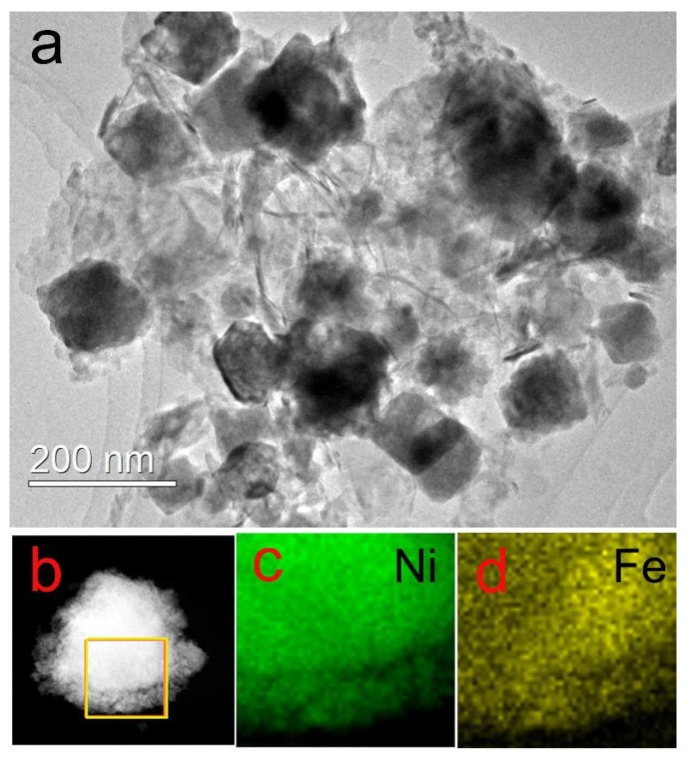
(**a**) TEM image of the NiFe/GO composite and (**b**–**d**) element mapping of the NiFe alloy.

**Figure 2:**
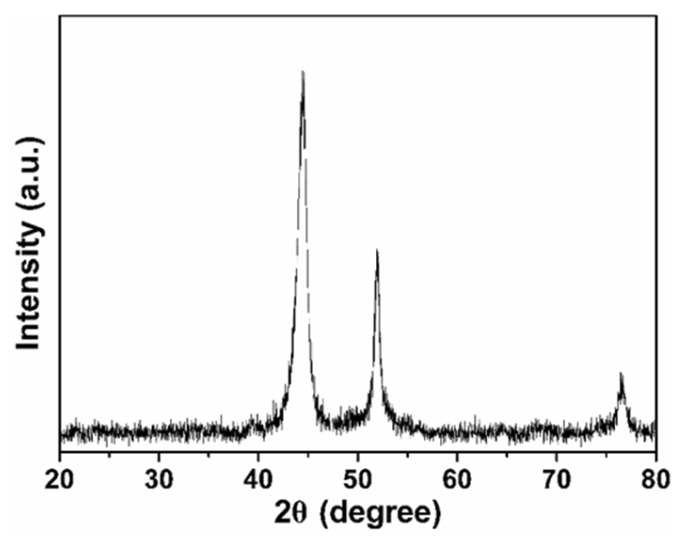
XRD pattern of the NiFe/GO composite.

The TEM image of the NiFe/GO composite is displayed in Figure 1. As shown in Figure 1a, NiFe alloy with diameter of about 100 nm are loaded on the GO. Figure 1b–d shows the element mapping of the NiFe alloy. It can be seen clearly that the Ni and Fe elements are evenly distributed in the NiFe alloy. This proves the successful preparation of the NiFe alloy. Figure 2 shows the XRD pattern of NiFe/GO to further investigate the crystalline structure. The diffraction patterns located at 44.5°, 51.9°, and 76.4° can be ascribed to the diffraction of the (111), (200), and (220) crystal planes of the NiFe alloy, respectively [41]. No peaks of GO could be found due to its small amount.

### 3.2. Electrochemical Measurements

**Figure 3:**
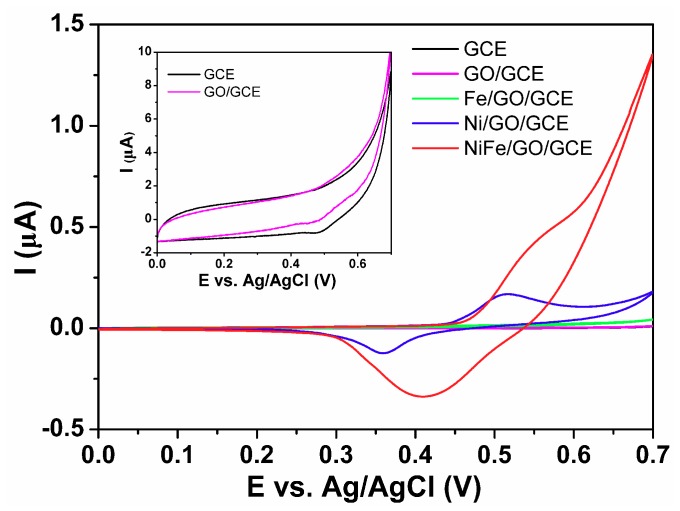
Cyclic voltammograms (CVs) of GCE, GO/GCE, Fe/GO/GCE, Ni/GO/GCE, and NiFe/GO/GCE in 0.1 M NaOH solution in the presence of 1.0 mM glucose at a scan rate of 10 mV/s. Inset: CVs of GCE and GO/GCE.

Figure 3 shows the typical cyclic voltammograms of the as-prepared GCE, GO/GCE, Fe/GO/GCE, Ni/GO/GCE, and NiFe/GO/GCE in 0.1 M NaOH solution in the presence of 1.0 mM glucose. It can be seen from Figure 3 that GCE, GO/GCE, and Fe/GO/GCE show very small current in the whole voltage range. Oxidation peaks between 0.50 to 0.55 V can be observed for Ni/GO/GCE and NiFe/GO/GCE, which can be ascribed to the oxidation of Ni^2+^ to Ni^3+^. The Ni^3+^ accounts for the oxidation of glucose [15]. When Fe was added to Ni to form the NiFe alloy, the current clearly increased. This indicates that the NiFe alloy has higher performance for electrochemical glucose detection.

**Figure 4:**
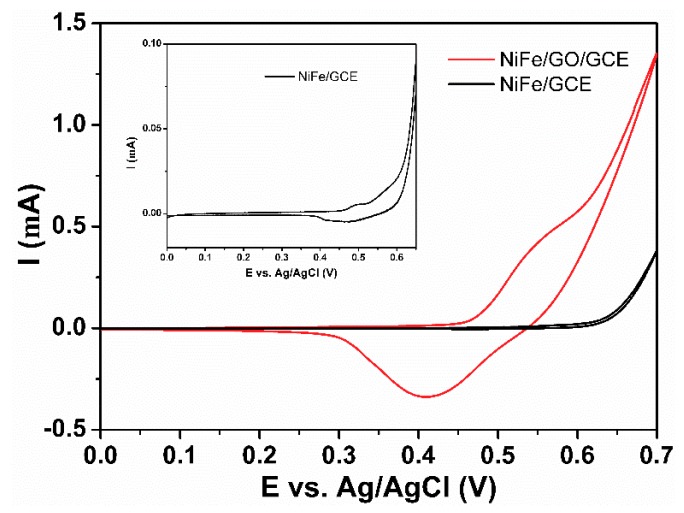
CVs of NiFe/GCE and NiFe/GO/GCE in 0.1 M NaOH solution in the presence of 1.0 mM glucose at a scan rate of 10 mV/s. Inset: CV of NiFe/GCE.

To illustrate the effect of GO, the performance of NiFe/GCE and NiFe/GO/GCE were investigated. As shown in Figure 4, NiFe/GCE has only negligible current and the redox peaks can be seen in the inset figure. NiFe/GO/GCE shows much larger current than that of NiFe/GCE, indicating GO is important in the composite electrode. The GO not only acts as the support to protect the NiFe alloy from aggregation, but also plays an important role for electronic transmission.

**Figure 5:**
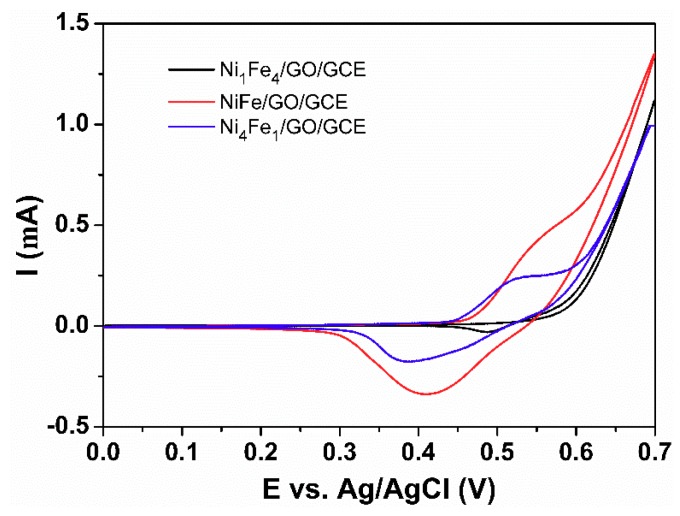
CVs of Ni_1_Fe_4_/GO/GCE, NiFe/GO/GCE, and Ni_4_Fe_1_/GO/GCE in 0.1 M NaOH solution in the presence of 1.0 mM glucose at a scan rate of 10 mV/s.

Furthermore, the effect of the ratio between the Ni and Fe elements were investigated. As shown in Figure 5, Ni_1_Fe_4_/GO/GCE shows the smallest current. With the increase of Ni amount, NiFe/GO/GCE shows the biggest current. Conversely, a greater amount of Ni decreases the current for Ni_4_Fe_1_/GO/GCE. So, NiFe/GO/GCE was used for further research.

**Figure 6:**
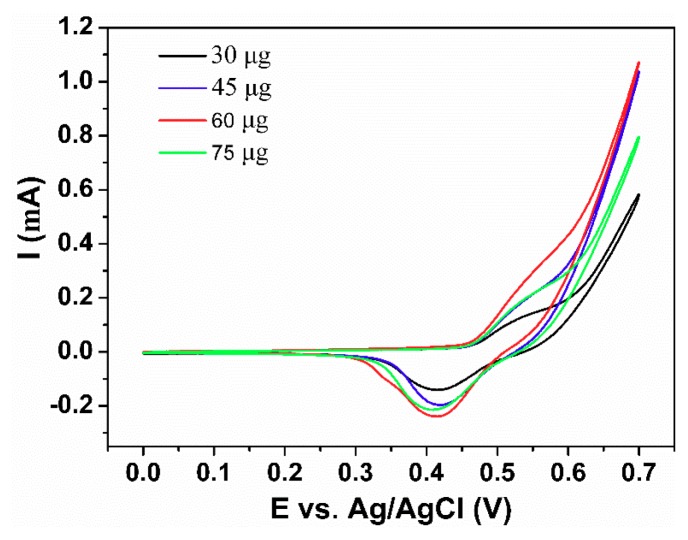
The effect of modification amount on the performance of the electrode.

In order to improve the electrocatalytic performance of NiFe/GO/GCE, the loading amount of NiFe/GO was studied. Figure 6 shows the cyclic voltammogram (CV) curves of NiFe/GO/GCE with different amounts of NiFe/GO loaded onto the GCE. It can be seen that the current increases gradually from the loading amount of 30 to 75 μg NiFe/GO. When the loading amount exceeds 60 μg, the current decreases. So, 60 μg is the optimum loading amount and is used in the later experiments. This phenomenon can be explained by the change of catalytic sites. At first, increasing the loading amount of the NiFe alloy increased the number of catalytic sites. However, an excessive loading amount limited the mass transfer process, leading to the decrease of current [19].

**Figure 7:**
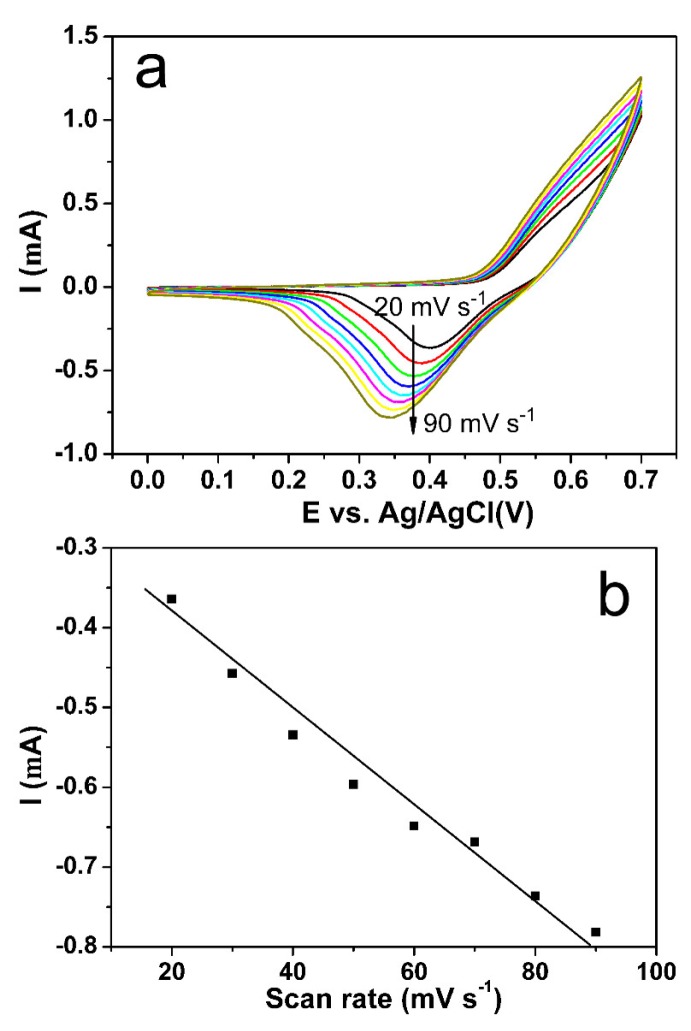
(**a**) Cyclic voltammograms of NiFe/GO/GCE at different scan rates from 20 to 90 mV/s in 0.1 M NaOH with 1.0 mM glucose. (**b**) Plot of cathode current vs the scan rate.

Cyclic voltammetric measurements were performed at increased scan rates to better understand the electrocatalytic properties of the NiFe/GO/GCE electrode for glucose oxidation. As demonstrated in Figure 7a, cathodic peak currents of NiFe/GO/GCE increased with the increasing scan rate. Figure 7b illustrates the relationship between the cathodic peak current and scan rate. It can be seen that the cathodic peak currents increase linearly with the used scan rates. This result manifested that the rate-determining step was surface reaction control rather than diffusion control, which is favorable for quantitative analysis.

### 3.3. Amperometric Response Towards Glucose Sensing

**Figure 8:**
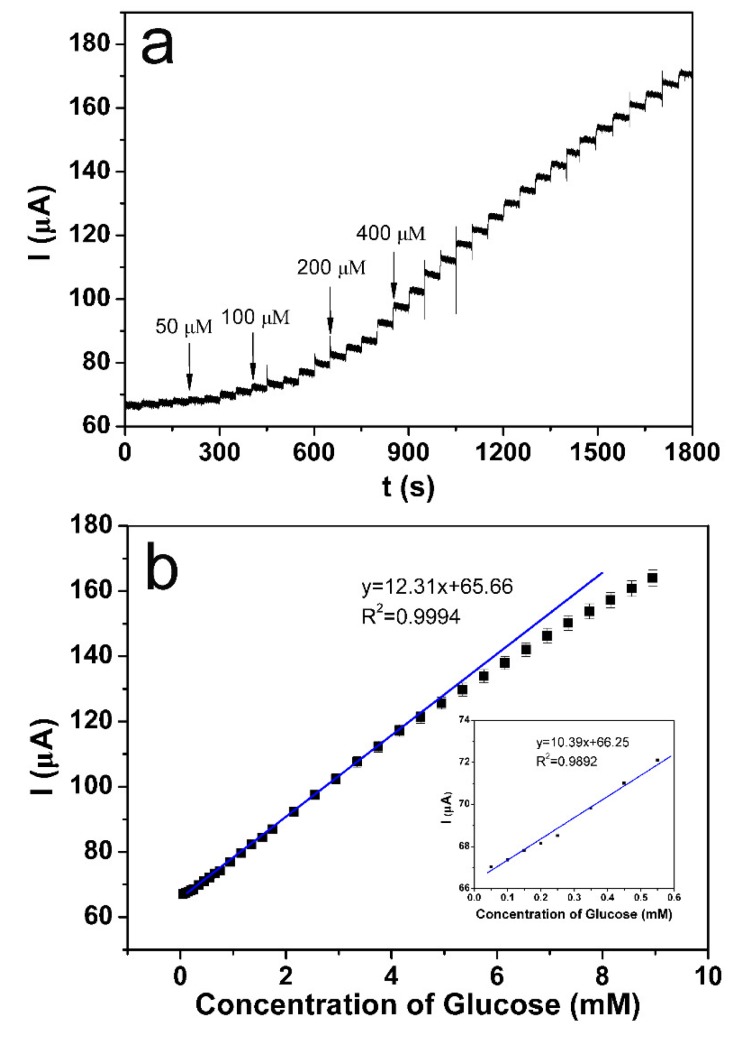
(**a**) Amperometric currents measured with the continuous addition of glucose with concentration from 50 to 400 μM at 0.55 V for NiFe/GO/GCE, and (**b**) the corresponding calibration curve. Inset: calibration curve at low concentrations.

Amperometric measurement was carried out to show the current response of the NiFe/GO/GCE electrode towards the continuous addition of various concentrations of glucose, which was carried out in vigorously stirred electrolyte. As shown in Figure 8a, the NiFe/GO/GCE electrode consistently generates a fast current response upon the addition of glucose with concentration from 50 to 400 μM. According to the amperometric current, the corresponding calibration curve was plotted in Figure 8b. It can be seen that the NiFe/GO/GCE sensor showed a wide linear sensing range, from 0.05 to 5 mM (R2 = 0.9994), and a sensitivity of 173 μA mM^−1^ cm^−2^. The limit of quantitation (LOQ) is 0.05 mM, while the limit of detection (LOD) is 9 μM at a signal/noise ratio of 3. At low concentrations, as shown in the inset in Figure 8b, the NiFe/GO/GCE sensor also showed a sensitivity of 148 μA mM^−1^ cm^−2^, which was close to that at high concentrations. As shown in Table 1, the performance of our sensor was compared with the previously reported sensors based on Ni materials. Our sensor is comparable to the previously reported sensors. The high sensitivity can be ascribed to the high electrocatalytic activity of the NiFe alloy and the excellent electronic transmission performance of GO.

### 3.4. Specificity and Reproducibility of the NiFe/GO/GCE Electrode

**Figure 9:**
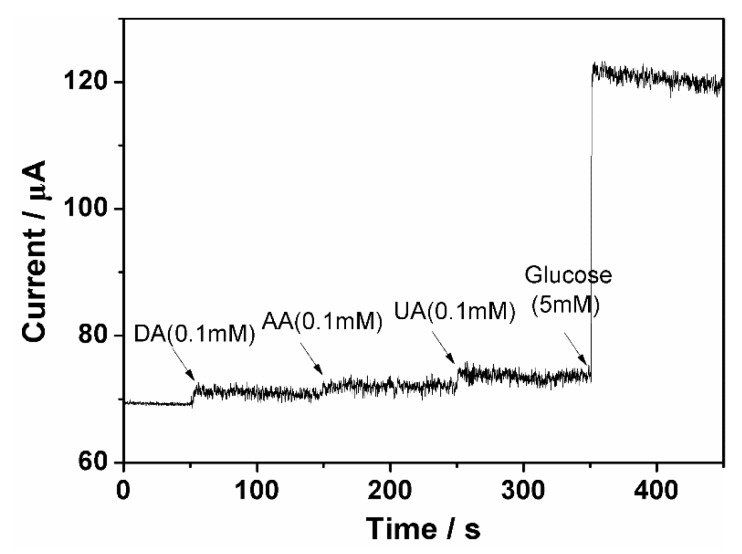
Amperometric response of the NiFe/GO/GCE electrode with interference by dopamine (DA), ascorbic acid (AA), and uric acid (UA) at physiological concentrations.

Dopamine (DA), ascorbic acid (AA), and uric acid (UA) coexist in human blood, which influences the electrochemical nonenzymatic glucose sensing. To investigate the effect of DA, AA, and UA on glucose sensing, an amperometric response test was carried out. Figure 9 shows the amperometric response current with the addition of DA, AA, UA, and glucose. It can be seen that the current generated by DA, AA, and UA in normal physiological concentrations are only 4.1%, 1.9%, and 2.6%, respectively, compared to that of glucose. This research demonstrates that the NiFe/GO/GCE electrode possesses specificity for glucose sensing and thus can be used in real-world sensing applications.

**Figure 10:**
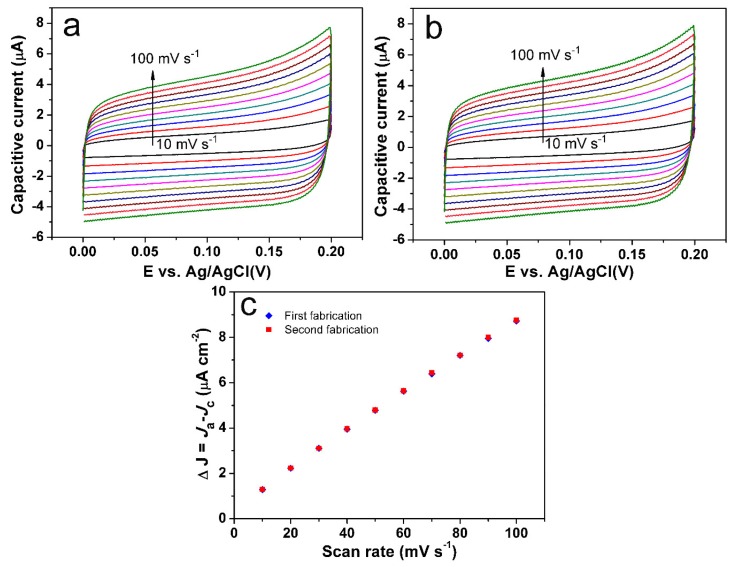
Cyclic voltammograms with different scan rates in the potential range of 0.0 V to 0.2 V vs. Ag/AgCl, where no Faradaic processes occur for the first (**a**) and second fabrication (**b**) of the electrode. Charging current density differences for the first and second fabrications (**c**) of the electrode are plotted against scan rates.

To check the reproducibility of our manufacturing operation, we fabricated two electrodes in the same conditions and tested their double-layer capacitance (Cdl). Cyclic voltammograms were carried out in 0.1 M NaOH solution at scan rates from 10 to 100 mV s^−1^ (Figure 10a,b). Then, the electrochemically active surface area was estimated by testing the capacitive current at non-Faraday regions at different scan rates, from which the double-layer capacitance (Cdl) was determined by plotting the △J = (J_a_ − J_c_) at 0.10 V versus Ag/AgCl against the scan rate, as shown in Figure 10c. The linear slope is equivalent to double the Cdl, which can be used to represent the electrochemically active surface area [48]. From Figure 10c, it can be seen that the two fabricated electrodes have almost the same linear slope, revealing the same electrochemically active surface area of the two electrodes fabricated in the same conditions. That is, our fabrication process is reliable.

### 3.5. Practical Applications

For practical analysis, the NiFe/GO/GCE electrode was used to detect the glucose concentration in human serum by amperometric measurement. Briefly, 20 μL of serum was injected into 15 mL of 0.1 M NaOH solution, and then the current response at +0.55 V on NiFe/GO/GCE was recorded. The recovery value was confirmed by standard injection of glucose with a known concentration to the above sample, then recording the current at +0.55 V. As listed in Table 2, the recovery values of all three samples were close to 100%, indicating good practical application potential of the NiFe/GO/GCE electrode.

## 4. Conclusions

In summary, a NiFe/GO/GCE electrochemical glucose sensor has been successfully fabricated. The addition of the Fe element into Ni nanoparticles to form NiFe alloy nanoparticles improved the electrochemical performance of the glucose sensor, which showed higher current than the Ni/GO electrode. In addtion, the GO not only acts as a support to load the NiFe alloy from aggregation, but also plays an important role for electronic transmission. The NiFe/GO/GCE showed best performance when the ratio of Fe to Ni was 1:1. It was determined that 60 μg is the optimum loading amount in the tested conditions. The NiFe/GO/GCE electrode exhibited excellent sensitivity (173 μA mM^−1^ cm^−2^) and a wide detection linear range (up to 5 mM). In addition, the NiFe/GO/GCE electrode shows high selectivity for glucose detection and can be applied to glucose detection in human serum. All results demonstrate that the NiFe/GO/GCE electrode is a promising candidate in the development of cheap, stable, and sensitive nonenzymatic glucose sensors.

## Figures and Tables

**Table 1:** Comparison of our sensor to other Ni-based sensors.

Materials	Sensitivity (μA mM^−1^ cm^−2^)	Linear Range	Reference
Pd−Ni/Si nanowires	190.7	0–20 mM	[42]
Ni−Pt	66.9	0.1–30.1 mM	[43]
Ni/Al LDH nanosheet	24.45	5 μM−10 mM	[44]
PVP–GNs–NiNPs–CS	103.8	0.1 μM–0.5 mM	[45]
PtNi–ERGO/GCE	20.4	Up to 35 mM	[46]
HollowPt–Ni–graphene	30.3	0.5–20.0 mM	[47]

**Table 2:** Amperometric detection of glucose in serum.

Sample	Concentration (mmol L^−1^)	RSD (%)	Added (mmol L^−1^)	Found (mmol L^−1^)	Recovery (%)
1	9.3	3.5	1	10.1	98
2	5.8	3.1	1	6.7	99
3	7.1	3.4	1	8.0	99
